# P02.72. A pilot investigation of alignment-based yoga for pediatric obesity

**DOI:** 10.1186/1472-6882-12-S1-P128

**Published:** 2012-06-12

**Authors:** K Hainsworth, K Salamon, S Stolzman, P Simpson, D Esliger, B Mascarenhas, X Liu, K Khan, B Fidlin, S Weisman

**Affiliations:** 1 Medical College of Wisconsin, Milwaukee, USA; 2 Children's Hospital of Wisconsin, Milwaukee, USA; 3 University of Saskatchewan, Saskatchewan, Canada; 4 Santosh Yoga, LLC, Wauwatosa, USA

## Purpose

Although exercise is a primary tool for weight reduction, recent findings of aberrant biomechanics in obese youth have raised concern over traditional exercise prescriptions. Given that injury and disability often act as barriers to physical activity (PA), particularly for those with increased weight, safe and appealing interventions are urgently needed. To that end, this study examined the benefits of a yoga intervention for obese adolescents.

## Methods

Adolescents referred to a pediatric weight management clinic (BMI > 95th percentile and ≥ 1 co-morbidity) were recruited to participate in an 8-week study involving bi-weekly, 60-minute Iyengar style yoga classes. All questionnaires and assessments of physical functioning were conducted immediately before and after the 8-week intervention. Assessments included prior experience and expectations, health-related quality of life (HRQOL), state anxiety, and functional limitations. Standardized assessments of participants’ physical abilities included push-ups, sit-ups, a step test, and sit to reach. PA levels were objectively assessed using a hip-mounted Actical accelerometer worn 7 consecutive days (pre and post-yoga).

## Results

Sixteen youth (11-17 years, M 13) attended at least 7 classes. Half reported experiencing pain in the 2 weeks prior to consent (usual pain intensity M 5.88 ± 2.30). Sit-to-reach improved (p < .05) from pre (M 6.20 cm ± 8.86) to post (M 8.83 cm ± 5.62) intervention. Across almost all domains, participant and parent reports of HRQOL significantly improved (p’s < .05). Self-reports of state-anxiety decreased (p < .05). Whereas time spent in Sedentary, Light and Vigorous PA did not change, time spent in Moderate intensity PA increased (p = .05) from pre- (M 21.82 min. per day ± 25.71) to post-yoga (M 27.26 min. per day ± 16.44) intervention.

## Conclusion

These preliminary findings are encouraging, and suggest that alignment-based yoga may be a safe and effective intervention for pediatric obesity.

